# The Fontan Circulation: When Net Flow Is Not the Whole Story

**DOI:** 10.1016/j.jacbts.2026.101703

**Published:** 2026-09-11

**Authors:** Vivek Muthurangu

**Affiliations:** UCL Centre for Translational Cardiovascular Imaging, University College London, London, United Kingdom

**Keywords:** cardiorespiratory effects, congenital heart defect, hemodynamics, single ventricle disease

**Figure:**
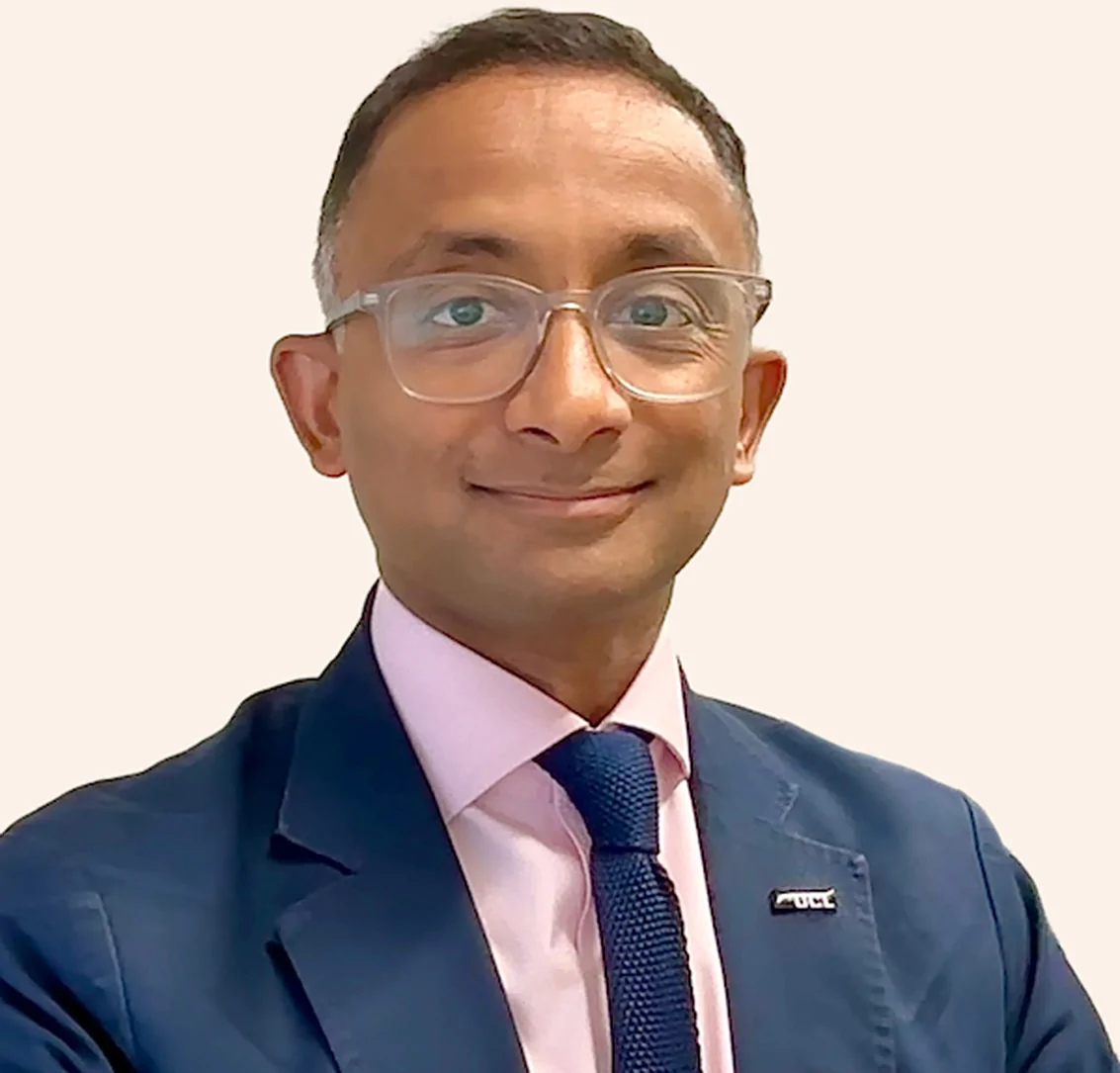

In this issue of *JACC: Basic to Translational Science*, Horvath et al^1^ elegantly dissect the effects of respiratory breathing patterns on systemic venous flow in the Fontan circulation. In contrast to the biventricular circulation, respiratory mechanics play an outsized role in the Fontan circulation because of the lack of a subpulmonary ventricle. The result is phasic variation in systemic venous flow during breathing, with many studies emphasizing augmented forward flow during inspiration because of reduced intrathoracic pressure. Historically, this observation gave rise to the appealing idea of an auxiliary “respiratory pump,” where inspiratory effort augments venous return and ultimately cardiac output. This physiological concept subsequently motivated interventions, including inspiratory muscle training,^2^ aimed at enhancing respiratory mechanics to improve Fontan hemodynamics.

However, the relationship between breathing and Fontan hemodynamics is more complex than this simple pump analogy suggests. Although inspiration increases forward flow, this is often entirely offset by reduced/reversed flow during expiration as intrathoracic pressure rises. This may explain recent observations that variations in respiratory pattern have surprisingly little effect on net venous return when integrated across the complete respiratory cycle.^3^

Nevertheless, there is evidence that specific venous flow patterns may be associated with poorer outcome, and thus further investigation is warranted.^4^ In their study, Horvath et al^1^ comprehensively interrogate the effects of breathing patterns on Fontan hemodynamics. They achieve this by leveraging real-time phase-contrast cardiac magnetic resonance (PCMR) to continuously measure inferior vena cava (IVC) flow during controlled alterations in breathing. A particular strength of their experimental design is the separation of 2 closely related components of respiration, namely respiratory effort and respiratory rate. Importantly, measures of respiratory rate and effort were derived directly from the PCMR data, allowing confirmation that manipulation of either variable did not significantly alter the other.

They showed that increasing respiratory effort increased both forward and retrograde IVC flow, which they appropriately attribute to greater variation in the transdiaphragmatic pressure gradient in deep compared with shallow breathing. The investigators also report an increase in the retrograde flow ratio with greater respiratory effort and suggest that this reflects a disproportionate effect on retrograde flow. However, this may simply be a mathematical consequence of the significantly smaller magnitude of retrograde flow. Consider an idealized example with baseline forward and retrograde IVC volumes of 150 mL and 15 mL, resulting in a retrograde flow ratio of 10%. If deep breathing equally increases both forward and retrograde flows by 10 mL, the resulting volumes of 160 mL and 25 mL yield a flow ratio of ∼16%. Conversely, if shallow breathing reduces both by 10 mL, the ratio falls to ∼4% (5 mL/140 mL). This simple example demonstrates that a change in the retrograde flow ratio does not necessarily indicate a preferential effect of respiration on retrograde flow.

The investigators also showed that manipulating respiratory rate yielded a distinctly different pattern, with faster breathing substantially reducing IVC forward flow per respiratory cycle and slower breathing having the opposite effect. Conversely, respiratory rate had either no effect on retrograde IVC flow (slow breathing) or increased retrograde flow per cycle (fast breathing). Although this may initially suggest a disproportionate effect of respiratory rate on forward flow, these findings also require cautious interpretation. Consider the idealized situation where inspiratory time changes proportionally with respiratory cycle length, but inspiratory flow rates stay the same. In this case, faster breathing will reduce forward volume simply because of a shorter inspiratory time, with the opposite occurring during slower breathing. In the study by Horvath et al^1^, respiratory cycle duration and IVC forward volume both increased approximately 1.6x during slow breathing, although this relationship was less exact during fast breathing (0.5x for cycle duration vs 0.65x for forward volume). Nevertheless, these findings are broadly consistent with the idea that apparent effect on forward volume may simply reflect changes in inspiratory duration rather than some other physiology. Intriguingly, this proportionality was not observed for retrograde flow. Despite shorter expiratory phases at higher respiratory rates, retrograde flow either stayed constant or increased. This potentially suggests that increasing rate actually disproportionally affects retrograde IVC flow.

Importantly, although respiration changed phasic venous flow patterns, it did not change net systemic venous return or cardiac output. This raises an important question: do phasic changes in IVC flow actually matter clinically? Horvath et al^1^ rightly note that increased retrograde IVC flow may result in repetitive spikes in hepatic venous pressure, as well as increased oscillatory shear stress. Furthermore, they suggest that these effects may contribute to Fontan-associated liver disease and other end-organ damage. To further investigate this, they also raise the interesting possibility of using PCMR-derived flow patterns to inform liver-on-chip experiments that could interrogate the underlying biology of Fontan-associated liver disease.

Of maybe greater importance, the investigators also highlight the energetic inefficiency that may result from bidirectional IVC flow, an important concept that warrants further discussion within the context of venous return. In Guyton's classic model,^5^ venous return is determined by the pressure gradient between mean systemic filling pressure (P_MSF_) and right atrial pressure, divided by the resistance to venous return (RVR)—Figure 1A. Importantly, because RVR has relatively limited capacity for acute modulation, increases in venous return during activities such as exercise rely largely on increasing P_MSF_. This is achieved through sympathetic reduction in venous capacitance, which recruits unstressed blood volume into the stressed compartment, which is the main determinant of P_MSF_.

**Figure 1:**
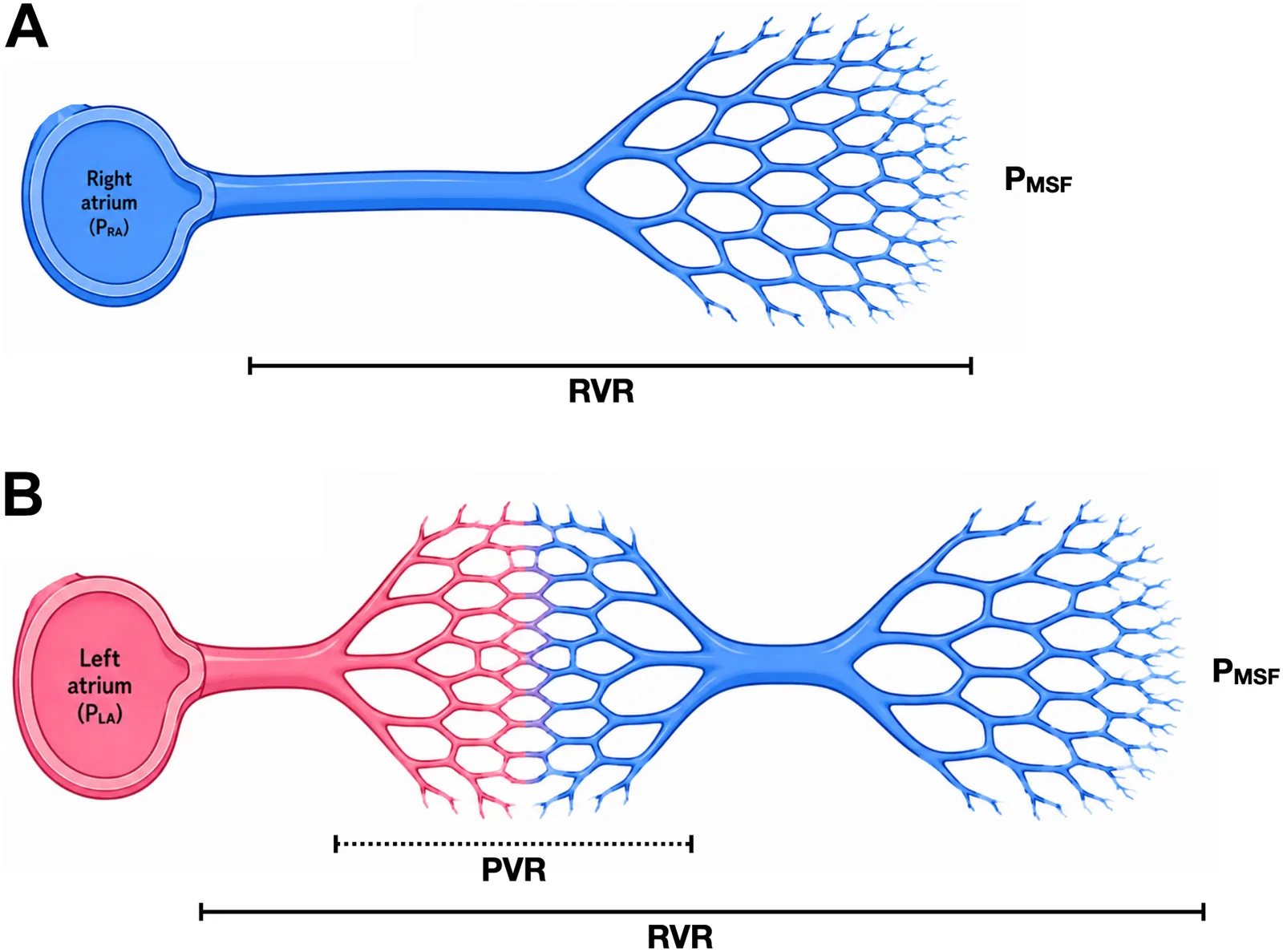
Venous Return in Normal and Fontan Circulation Comparison of venous return pathways in (A) normal and (B) Fontan circulation. P_MSF_ = mean systemic filling pressure; PVR = pulmonary vascular resistance; RVR = resistance to venous return.

In the Fontan circulation, this relationship is fundamentally altered because RVR now additionally includes the pulmonary vascular resistance, whereas right atrial pressure is replaced by left atrial pressure—Figure 1B. Because the resistances of the systemic venous system and pulmonary vasculature are of similar magnitudes, the effective RVR approximately doubles.^6^ This would significantly reduce venous return and cardiac output without compensation, which is achieved by increasing stressed volume both through reduced venous capacitance and chronic expansion of total blood volume. Although this maintains adequate venous return at rest, the resulting increase in P_MSF_ comes at the cost of systemic venous congestion and reduced ability to further augment venous return. This contributes to both the exercise intolerance and end-organ damage that is so characteristic of patients with Fontan.

It is within this framework that we can better understand the observations of Horvath et al.^1^ Although resistance is often considered a geometric property of the vasculature, an alternative view is that it is a measure of irreversible energy loss because of viscous dissipation. It is well recognized that retrograde and oscillatory flow can promote flow collision, turbulence, and vortices, all of which increase irreversible energy loss.^7^ This results in a further increase in effective RVR, over and above that seen in a Fontan with minimal retrograde flow. In this situation, maintaining the same net venous return would require an increased P_MSF_, further exacerbating venous congestion and reduced ability to augment venous return. In fact, at sufficient magnitude, retrograde flow could impose a hemodynamic burden analogous to anatomical narrowing of the Fontan pathway. Thus, respiratory modulation of venous flow patterns is potentially of great importance, even if net venous return is unchanged.

In conclusion, Horvath et al^1^ demonstrate that breathing patterns and venous flow characteristics are intimately linked in the Fontan circulation. Further work is needed to understand how these relationships interact with physiological states that increase IVC flow, such as exercise and digestion. Nevertheless, this study provides an important foundation for future investigation of this clinically important and fascinating physiology.

## Funding Support and Author Disclosures

The authors have reported that they have no relationships relevant to the contents of this paper to disclose.
